# A study of the efficacy and cost-effectiveness of MRSA screening and monitoring on surgical wards using a new, rapid molecular test (EMMS)

**DOI:** 10.1186/1472-6963-7-160

**Published:** 2007-10-03

**Authors:** Katherine J Hardy, Ala Szczepura, Ruth Davies, Andrew Bradbury, Nigel Stallard, Savita Gossain, Paul Walley, Peter M Hawkey

**Affiliations:** 1 West Midlands Public Health Laboratory, Health Protection Agency, Heart of England NHS Foundation Trust, Bordesley Green East, Birmingham, B9 5SS, UK; 2 Department of Immunity and Infection, University of Birmingham, Edgbaston, Birmingham, B15 2TT, UK; 3 Department of Research and Development, Heart of England NHS Foundation Trust, Bordesley Green East, Birmingham, B9 5SS, UK; 4 Medical School, University of Warwick, Coventry CV4 7AL, UK; 5 Warwick Business School, University of Warwick, Coventry, CV4 7AL, UK

## Abstract

**Background:**

MRSA is a significant contributor to prolonged hospital stay, poor clinical outcome and increased healthcare costs amongst surgical patients. A PCR test has been developed for rapid detection of MRSA in nasal swabs. The aims of this study are (1) to estimate the effectiveness of screening using this rapid PCR tests vs culture in reducing MRSA cross-infection rates; (2) to compare the cost of each testing strategy, including subsequent health care costs; and (3) to model different policies for the early identification and control of MRSA infection in surgical patients.

**Methods/Design:**

The study is a prospective two-period cross-over study set in 7 surgical wards covering different surgical specialities. A total of 10,000 patients > 18 years will be tested over 16 months. The only difference between the two study periods is the method used for the detection of MRSA in each ward (rapid v conventional culture), with all other infection control practices remaining consistent between the arms. The study has been designed to complement routine practice in the NHS. Outcomes are MRSA cross-infection rates (primary outcome) and need for antibiotic therapy and MRSA-related morbidity. Parallel economic and modelling studies are being conducted to aid in the interpretation of the results and to evaluate the cost-effectiveness of the rapid PCR screening strategy.

**Discussion:**

This paper highlights the design, methods and operational aspects of a study evaluating rapid MRSA screening in the surgical ward setting.

## Background

MRSA is a major cause of nosocomial infection in hospitals throughout the world. Rates of infection and colonisation vary substantially between different hospitals both within and between different countries. The UK has one of the highest rates within Europe and the need for improved intervention has been highlighted by experts in infection control [1]. In particular, MRSA is a significant contributor to prolonged hospital stay, poor clinical outcomes and increased healthcare costs amongst surgical patients [2]. The number of death certificates in England and Wales mentioning MRSA has increased from 734 in 2001 to 1,629 in 2005, with an increase of 39% from 2004 to 2005 [3]. Despite there being many reports in the literature of localised successful control of MRSA within the hospital setting, world-wide the incidence of MRSA continues to rise outside of areas such as the Netherlands and Scandinavia, where intensive control has managed to maintain extremely low rates of MRSA [4].

The major method for instituting control is the microbiological identification of patients either colonised or infected with MRSA, followed by isolation of these patients to prevent cross-infection and decolonisation treatment with topical nasal mupirocin and skin disinfection to eliminate carriage [5]. The influence of interventions including active screening and isolation policies on the rates of colonisation and infection caused by MRSA has been comprehensively reviewed recently [1]. This review concluded that, most, intervention studies to date have been poorly designed and poorly controlled, with only one prospective cross-over cohort study, and two prospective cohort studies which were less rigorously controlled. In some studies nurse cohorting in bays was practised on wards and these studies appeared to show a positive effect [6]. The review also included some comprehensive mathematical modelling to try to understand the variables affecting MRSA colonization rates, and concluded that improvements in detection rates and/or isolation capacity should lead to reduced rates of endemic MRSA. However, the review group also found inadequate economic evaluation in all of the studies undertaken, together with a failure to take adequate account of the influence of confounding variables. Their recommendations were that, although current isolation measures appear to have some effect on maintaining lower levels of MRSA colonisation in the UK, further research should be undertaken to provide improved evidence on the clinical effectiveness and cost-effectiveness of MRSA screening and isolation interventions.

*Staphylococcus aureus *strains that express high-level resistance to methicillin (and related agents such as flucloxacillin) produce an additional low affinity penicillin binding protein (PBP2a) (i.e. not inhibited by beta-lactam antibiotics) encoded by the *mec*A gene. Detection of the *mec*A gene in cultures is a reliable surrogate marker for methicillin resistance. Until comparatively recently MRSA detection has largely relied on conventional culture methods on agar plates, which can take 48–72 h to obtain a result [7]. The addition of a broth enrichment step has been shown to improve the sensitivity, but extends the time to result to at least 72 h. However, the length of time to obtain a result limits the usefulness of both these methods, especially with the increasingly short length of hospital stays.

In recent years a number of different molecular methods for the rapid detection of MRSA have been described. The majority of these rely on multiplexed PCR primers to detect genes which identify strains as *S. aureus *(*nuc *is frequently used) and genes which are involved in the expression and mediation of methicillin resistance (*mecA *&*fem*) [8,9]. Such methods are generally only applicable to the identification of purified cultures of staphylococci, and therefore they cannot be used directly on samples [10]. Furthermore, these assays are unable to provide a reliable result from nose and other swabs due to the presence of coagulase negative staphylococci carrying *mecA *giving rise to a false positive result if MSSA are also present. Recently a new rapid test (the IDI-MRSA test) has been developed which is specific for detecting MRSA in nasal swabs. The PCR primers amplify the right hand extremity of the SCC*mec *genetic element, which contains the *mecA*, and *orfX*, an opening reading frame, which is specific for *S. aureus *[11]. An early evaluation on 288 patients has reported a 91.7% sensitivity with a processing time of 1.5 h [12].

A review commissioned by the NHS Health Technology Assessment (HTA) Programme has identified a need for accurate economic assessments of the costs of MRSA control, including the opportunity costs [13]. A recent Dutch study has considered cost and shown that implementation of an aggressive search and destroy MRSA policy within one large University Medical Centre cost €0.28 million per annum. Although an economic analysis suggests that failure to institute this policy would have resulted in greater expenditure, this was not based on prospective study data [14]. An extensive survey of the surveillance data reported in Wales supports the assertion that there are substantial costs associated with MRSA infection, with a deleterious impact on healthcare outcomes on at least half of the patients found to be infected [15]. Much of the attributable cost relates to increased length of stay, along with treatment with a variety of antibiotics, enteral feeding, and intensive care [2].

This paper describes the design, methods and operational aspects of a study investigating the efficacy and cost-effectiveness of rapid MRSA screening and monitoring on surgical wards.

The study has four aims:

1. To measure the impact of screening using rapid MRSA tests versus the most commonly used current test (i.e. culture plate), both in terms of reduction of MRSA cross-infection and improved clinical management and health service resource use.

2. To establish the reliability, turn around time, skills requirement for IDI test in the diagnosis of MRSA versus the most commonly used current test.

3. To assess the cost effectiveness of a screening strategy for MRSA in surgical patients using rapid IDI tests and infection control strategies versus current national MRSA control recommendations, and to model the costs and benefits of different protocols for the early identification and control of MRSA infection in these patients.

4. To produce generalisable protocols and guidelines, in the context of laboratory provision and MRSA levels across the UK, on the use of IDI in the management of MRSA cross infection in surgical patients.

## Methods/Design

### Study Setting and Population

The study is set in a large teaching hospital with over 1,200 beds, providing services to a large population with a wide range of socio-economic, ethnic and age characteristics. All patients admitted to the 7 surgical wards are included in the study. The seven wards include general surgery (2), vascular (1), thoracic (1), ENT (1), trauma and orthopaedics (2) and urology (1). The total capacity of the wards ranges between 20 and 34 beds, and in total is 195 beds; with an average length of stay on the wards of 4.5 days. All of the wards have a limited number of single rooms (2 – 5), none of which have ensuite facilities.

### Inclusion and Exclusion Criteria

All patients aged 18 years plus who have a planned or emergency admission to a surgical ward, including all medical patients and internal transfers are included in the study.

### Overall study design and approach

The study is being carried out as a prospective cluster randomised two-period cross over study. A cross over study design allows a more rigorous means of measuring effectiveness than a simple prospective comparative study between wards. The only difference between the two study periods is the method used for the detection of MRSA in each ward, all other infection control practices are consistent between the wards. The aim of the study is to compare: (i) 'molecular' wards: patients are screened using the rapid IDI-MRSA test; and (ii) 'technical' wards: patients are screened using conventional culture. Results from the IDI_MRSA test are fed back within 2 hours of receipt of the samples to the molecular wards and within 2 days to the technical wards. One study arm contains 4 wards (Wards A), general surgery (1), trauma and orthopaedic (1), ENT (1) and thoracics (1), whilst the other arm contains 3 wards (Wards B), general surgery (1), trauma and orthopaedics (1) and urology (1). In the first period of the cross-over design, wards A were assigned to the molecular group and wards B to the technical group, with the assignment reversed for the second period.

Prior to commencement, educational sessions were held and information leaflets provided to all health care professionals working within the surgical directorate to provide them with information about the study and emphasise the importance of the work. Patient information leaflets, designed to ensure individuals are aware of necessary procedures for themselves, staff and visitors are provided.

Ethical approval was received from East Birmingham Ethics Committee. Written consent from study participants is not required as screening patients for MRSA is hospital policy. An initial two month pilot period was used to allow resolution of all operational and technical problems. This was followed by two 8 month cross-over periods, with one month follow up of study patients at the end of the final period. Wards A were allocated to the molecular arm of the study in the first period and Wards B to the technical arm.

### Sampling

The study design uses only nasal samples for MRSA screening, for several reasons. Firstly, and most importantly, at the start of the study all published work evaluating the IDI-MRSA assay had used only nasal samples [11]. Secondly a nasal sample is easily obtainable for large scale screening, ensuring comprehensive coverage. Additionally, in previous studies nasal samples have been shown to have a high sensitivity (84%) [16]. Screening swabs are taken from all patients on admission and then if the sample is negative the patient is screened every four days until discharge, and upon the day of discharge. Regular sampling at 4 daily intervals enables the implementation of infection control procedures in a timely manner and the detection of cross infection. Additional samples, for example blood cultures and wound swabs are sent on clinical suspicion as is normal hospital policy.

All samples from the molecular wards are collected by a research nurse at 8 am and 2 pm, Monday to Friday and once daily at 1 pm on a Saturday and Sunday. Samples from the technical wards are transported to the laboratory using the hospital portering service which operates during the day 7 days a week.

### Rapid communication of results to the wards

All results, both positive and negative, are made available on the laboratory reporting system to which all ward staff have access via a computerised reporting system, immediately upon completion of the test. In addition, all positive MRSA results, both rapid and conventional are telephoned to nurses on the wards, with the time and the person receiving the result documented.

### Laboratory protocols

The traditional method for the identification of MRSA is by culturing swabs directly onto agar plates. A recent review has concluded that use of Baird-Parker containing ciprofloxacin agar plate without broth pre-enrichment is the most cost effective screening strategy [7]. However, due to the recent emergence of ciprofloxacin sensitive community MRSA strains, we have chosen not to use this method [17]. Furthermore, since this publication several chromogenic agars have been developed which offer a high degree of sensitivity and specificity [18]. Therefore the methodology chosen was to use direct inoculation onto MRSA ID, a chromogenic media, (Biomerieux, Marcy l'Etoile, France), followed by confirmation using DNase and slide agglutination tests (Oxoid, Basingstoke, UK) for identification of *Staphylococcus aureus *followed by full sensitivity testing using BSAC methods [19].

The samples from the molecular wards are always received in batches; on week days two batches of samples are received, one at 8 am and another at 2 pm, whilst at the weekend only one batch is processed. On receipt of the samples all swabs are immediately inoculated directly onto chromogenic media and then using the same swabs the DNA is extracted using the BD GeneOhm protocol. The primers and molecular beacons used are supplied by the manufacturer (Becton Dickinson, NJ, USA) and the real time amplification of DNA is run on Smartcyclers™ (Cepheid, CA, USA). All agar plates are read after overnight incubation, and confirmed using DNase and slide agglutination followed by full sensitivity testing using BSAC methods [19].

In cases of discrepant results between the PCR and culture, all direct culture plates are incubated for an additional 24 h before being reported as negative. Additionally all swabs which are positive by the PCR method are placed in brain heart infusion broth (Oxoid, Basingstoke, UK) on the day the PCR is completed, incubated overnight, sub-cultured onto chromogenic agar and confirmed as above. This additional step was added because it is known that although direct culture is the preferred method of detecting MRSA for operational reasons in the UK i.e. due to cost and time implications, it is not the most sensitive.

Swabs from the technical wards are processed throughout the day, Monday to Friday and on Saturday mornings. The swabs are inoculated directly onto chromogenic agar and confirmed as previously described. All MRSA isolates, from both the technical and molecular wards are saved on protect beads and stored at -80C. The first isolate from each patient is epidemiologically typed using staphylococcal interspersed repeat unit (SIRU) typing as previously described and all results entered into Bionumerics™ [20].

### Patient follow up

Prior to starting the study the length of stay on each of the surgical wards was obtained and was shown to be an average 4 days. Therefore it is also necessary to capture data regarding any surgical site infections post discharge. On discharge from the study ward each patient receives a letter and a questionnaire to be completed by their general practitioner (GP) in the event that they visit their GP for treatment of their surgical site wound. The questionnaire collects follow up information on whether any antibiotics have been prescribed by the GP and the state of the surgical site wound. In addition, the consultants who review the patient in outpatients are asked to complete the same questionnaire if they identify a surgical site infection.

### Patient information and communication

Upon admission to a study ward all patients receive an information leaflet about the study; detailing what MRSA is, why it is important to screen for MRSA and why we are undertaking the study. A research nurse is available to provide further information and answer questions. If a patient is found to be MRSA positive during their stay they are given a second information leaflet answering a number of questions regarding colonisation with MRSA, and in addition they receive a third information sheet on the correct use of the decolonisation treatment. Upon discharge from the ward each patient receives a final information sheet as well as a questionnaire to be completed by their GP following any visit regarding their surgical site. For short stay patients who are discharged before their admission result is available, a letter is sent to them at home informing them that MRSA had been isolated from a screening sample. This is accompanied by an information sheet with contact details for a research nurse they were able to phone for information and reassurance regarding the MRSA. If the patient is discharged to a nursing home the community infection control nurses is contacted and liaises with the nursing home.

### Implementation of infection control measures

The infection control procedures implemented are standardised across all wards regardless of the method of testing for MRSA. Upon identification of a patient being colonised with MRSA the result is telephoned to the ward. If space is available, the patient will be placed in a side room on the ward. If a side room is not available, or if the patient needs to be observed, the individual will nursed on the open ward. When operationally possible wards with several patients colonised with MRSA will nurse these patients together in one bay (cohort nursing) and dedicate a specific nurse to care for the patients. For all these patients an isolation precautions barrier sign (yellow and black) is placed by the bed side; gloves, aprons and alcohol gel are placed at the end of the bed and the patient is commenced on decolonisation treatment. Decolonisation treatment consists of five days of aquasept skin wash, containing triclosan and mupirocin nasal cream, followed by two days of no treatment and then re-screening of the patient. For patients who have strains that are resistant to mupirocin, Polyfax, containing polymyxin B and bacitracin, is used as the alternative. Additionally all patients who are colonised with MRSA receive a surgical review, this is aimed at establishing whether the surgical procedure is necessary immediately or can be delayed. In the former case the surgical prophylaxis is changed to teicoplanin (standard prophylaxis is cefuroxime and metronidazole), but if it is possible to delay surgery the patient undergoes a course of decolonisation treatment as detailed above, either at home or within the hospital.

The implementation of all infection control measures is audited within 12 hours of a patient being identified as being colonised with MRSA followed by continuous monitoring during their admission by the study research nurses. All measures are documented, and any failures to comply with these are both documented and rectified.

### Data collection

A comprehensive data set is being collected on all study patients. Data is entered directly by the research nurses onto laptops on the wards using a specifically modified Access database and then uploaded onto a secure server. Information collected includes patient ID, demographic data, risk factors, antibiotic usage, bed movements, length of stay, and treatment outcomes. For patients who are colonised with MRSA additional information is collected regarding implementation of infection control measures and decolonisation treatment.

Laboratory data collated includes patient ID, date and time sample taken and received by lab, and the IDI_MRSA and culture results.

### Data analyses

Analysis will be based on all individuals presenting for admission to the 7 surgical wards (intention to treat), including emergency and planned admissions. Numerical and graphical summaries of all the data will be compiled, including a detailed description of missing data at the ward and individual level.

Sensitivity and specificity will be estimated for PCR and culture tests. Receiver operating characteristic (ROC) curves are not appropriate because this is not a quantitative test. However, the positive predictive value (PPV) and negative predictive value (NPV) will be calculated. We will examine test replicability (i.e. precision) which reflects the variance in a test result that occurs when the test is repeated on the same specimen. Test reliability (e.g. failure rates) and laboratory turn-around time will also be compared.

The main clinical outcome measures will be MRSA cross-transmission rates, antibiotic therapy and MRSA-related morbidity. Secondary outcome measures will include length of in-patient stay, ITU episodes, a final outcome recorded at discharge, and MRSA infection at the outpatient follow-up clinic. Measures of resource use will include the cost of hospital episode and any re-admissions for MRSA.

The numbers of patients becoming infected, the cross-transmission rates and the numbers of MRSA-related deaths within each ward will be compared for the molecular and technical periods. The main analysis will focus on ward by period level data summaries. It seems likely that the number of new MRSA cases observed in each ward in each period will follow a Poisson distribution with mean depending on the size and type of the ward and whether the molecular or technical testing is being used in that period. A non-linear repeated measures random-effects analysis will be used to compare the mean number of cases, rate of cross-infection and number of MRSA related deaths. Ward by period interaction-level covariates could be included in this analysis. Depending on the observed rate of MRSA colonisation or infection and admission to the different wards, a normal approximation to the Poisson distribution may be appropriate, enabling linear modelling techniques to be used.

Further analyses will use linear or non-linear mixed effect models to assess the effects patient-level covariates. Estimates of the difference between the molecular and technical periods adjusted for the effect of covariates will be obtained.

Health service resource use will similarly be compared.

### Sample size

Given the limitations on published data available to inform the sample size estimate at the outset of the study, it was decided, in the light of operational constraints, to monitor MRSA colonisation and infection rates in seven wards for eighteen months, with a cross-over from molecular to technical testing or vice versa after nine months. Admission rates to the wards led to an estimated total of 10,000 patients being included in the study, with 5000 being assigned to each of the two periods.

Assuming that 5% of patients are MRSA colonised on admission to the wards, with a transmission rate in the technical wards of 0.5, a total of 125 new cases of infection in the control arm would be expected.

The power of the study can be calculated assuming that the number of new cases follows a Poisson distribution and using the normal approximation to the Poisson. If, for the purpose of the power calculation, it is assumed that admission rates and MRSA colonisation rates on admission are the same for all seven wards and in both periods, and that and MRSA transmission rates are the same for all wards using molecular or technical testing, a total of 10,000 admitted patients would mean that the study had 90% power to detect a reduction in the transmission rate from 0.5 to 0.3, corresponding to a drop in the expected number of new cases in the molecular wards from 125 to 75.

### Economic analysis

MRSA transmission may have a range of direct cost consequences to healthcare. The costing study will estimate differences in the cost of resources used in the molecular and technical wards. The economic analysis will compare resource use (costs) with any measurable changes in health outcomes (benefits). The health outcomes (or endpoints) will be measured in terms of changes in MRSA transmission and subsequent health outcomes. Any uncertainties in the cost and outcomes data will be incorporated into a sensitivity analysis.

The cost of each test will be determined through observation in the laboratory, once the learning curve is over and routine use of the technology is established. Maximum throughput for each method will be estimated. Test costs will include: labour (skill levels will be assessed for particular tasks); consumables; capital (converted into an annual equivalent cost and apportioned to tests based on annual throughput); maintenance costs; and overheads.

In both types of ward, other NHS costs related to patient care will be estimated. These will include resources associated with length of stay/ITU episodes, prescribing costs, and for MRSA positives post discharge care costs, including subsequent admissions for MRSA. Costs of any other relevant treatment will also be recorded. The use of services will be costed from a variety of sources, including the finance departments of the hospital and national sources [21]. Hospital notes and records will also be audited for information on service use.

The appropriate technique of economic evaluation will depend on the results of the study [22]. The simplest eventuality would be where the least expensive intervention is found to be better on at least one outcome measure and no worse on any other i.e. dominant. Another is where two interventions have the *same *outcomes (e.g. MRSA cross-infection rates), in which case the economic evaluation required when comparing the two is a cost-minimisation analysis. However, where an intervention is clearly better in terms of outcome but also more costly, a different approach is required. One accepted method is to compare the different interventions in terms of a single outcome measure identified as clinically important. The primary outcome measure meets this requirement. Therefore, the cost per unit improvement in MRSA transmission rate will be used to provide an estimate of overall cost-effectiveness of a screening strategy for MRSA in surgical patients using rapid IDI tests and infection control strategies.

### Modelling

Modelling will be undertaken of the policies for early identification and control of MRSA colonisation and infection in surgical patients. The purpose of this modelling is to understand and describe the transmission of infection within hospital wards in order to evaluate the effect of different policies with respect to speed of detection of infection and the means of prevention of transmission. We can for example compare the impact of using the rapid IDI-MRSA test on a theoretical ward with good isolation and cohort facilities as compared to one without.

There have been several previous models of MRSA transmission, including Cooper et al. who criticised previous models of MRSA transmission because they do not account for its persistence in hospitals [1,23]. They themselves produced compartmental deterministic and stochastic models to describe the transmission of MRSA with and without patient isolation. These models depict the movement between community and hospital and assume that all infection is transmitted directly from patient to patient, with all uninfected non-isolated patients being equally likely to be infected. On a detailed micro-level this is rather simplistic as, in practice, patients who are in close proximity to patients colonised with MRSA, or are admitted to intensive care are at greater risk of acquiring with MRSA [24].

Our modelling approach is to use agent based simulation which is a relatively new technique in this area for which user friendly software is now available. In agent-based simulation, each patient or carer can be an agent and can carry their own parameters for transmission or infection, which may depend on their location, hygiene levels and other factors.

In order to prime the model with data, we are using the most recent information from the literature concerning the mode of transmission of MRSA. Furthermore, as the modelling exercise is taking place towards the end of the research project we are able to take advantage of the data collected in the primary research.

The results from the model will enable us to evaluate different hospital policies to reduce the spread of MRSA. They will show the effectiveness in terms of a reduced colonisation or infection rate and the potential benefits of rapid testing. Finally we aim to show how powerful, flexible and effective agent based simulation is, in this context.

## Discussion

We have described the protocol and initial stages of the conduct of a large scale UK prospective cross-over study designed to evaluate the use of a rapid PCR testing for MRSA screening in patients admitted to 7 surgical wards in a large teaching hospital. To date, similar intervention studies have been poorly designed and controlled, with inadequate economic evaluation [25]. Most of these studies have been conducted in a single ward, predominantly ICUs. The main challenges in studying this area include ensuring that there are no changes to the hospital infrastructure and that policies and procedures remain unchanged during the study period, as well as the need to adequately consider all confounding variables.

The present study will be of importance in establishing the costs and benefits of introducing rapid MRSA screening in a standard NHS hospital setting which have limited single rooms. Also, due to high occupancy rates and dependency based nursing, cohort nursing in bays for patients who are colonised or infected with MRSA may not always be possible, despite best efforts of staff. This represents the situation in most hospitals in the UK. During the initial setting up of the study rapid transmission of results direct to a key nurse on the molecular wards using a text bleep system was envisaged. However, obstacles were encountered in maintaining confidentiality when texting patient identifiable information and in staff responsibility for the bleep. Individual nurses were reluctant to carry the bleep which was therefore often left on the nurses' workstation and therefore results were not always acted upon. Instead results were made available on a computerised laboratory reporting system accessible to all ward staff and positive MRSA results telephoned directly to nurses on the ward.

The study is currently completing the recruitment phase and is running well. Rapid MRSA screening has been well received by both patients and clinicians, following an initial reluctance of nursing staff to take on additional tasks. Much effort has gone into maintaining the profile of the study across all staff on the surgical wards, in the laboratory, and in the hospital infection control team.

The main strengths of the study are its prospective controlled design, the integral economic evaluation, the use of mathematical modelling and epidemiological typing to accurately identify transmission events and to enable the determination of optimum ways of reducing transmission, and the general applicability of the final findings to the current infrastructural context (e.g. isolation capability) of the NHS.

## Competing interests

PMH, AS and AB have received funding for the project from the Department of Health and Becton Dickinson. The authors declare that there are no other competing interests.

## Authors' contributions

KH participated in the design and coordination of the study, and drafted the manuscript. PMH and AS conceived the study, participated in its design and helped to draft the manuscript. AB, SG, RD and PW participated in the design and coordination of the study. NS provided statistical advice and helped draft the manuscript. All authors read and approved the final manuscript.

## Pre-publication history

The pre-publication history for this paper can be accessed here:


